# Cosmetic Appeal, HRQoL, and Effectiveness of Simple and Pseudotesticular Techniques of Orchidectomy in Prostate Cancer

**DOI:** 10.1155/2021/9968570

**Published:** 2021-11-26

**Authors:** Ijeoma N. C. Chibuzo, Augustine O. Takure, Olayiwola B. Shittu, Linus I. Okeke

**Affiliations:** ^1^Department of Surgery, University College Hospital, Ibadan, Oyo State, Nigeria; ^2^College of Medicine, University of Ibadan, Ibadan, Oyo State, Nigeria

## Abstract

**Introduction:**

Orchidectomy is the most cost-effective means of hormonal therapy for locally advanced or metastatic prostate cancer (LAMP). However, cost-effectiveness should not detract from health-related quality of life (HRQoL) considerations. Bilateral simple orchidectomy (BSO) has been linked to negative psychometric deficits from an empty scrotum. This study compared the HRQoL, therapeutic efficacy, and cosmetic appeal of BSO with endogenous pseudotesticular techniques of bilateral subcapsular orchidectomy (BSCO) and bilateral-epididymal-sparing orchidectomy (BESO). *Research Design*. Nigerian patients with LAMP were randomised into three surgical arms: BSO, BSCO, and BESO. Expanded Prostate Cancer Index Composite-26 HRQoL and sociodemographic questionnaires were administered before and three months after orchidectomy. Serum testosterone and PSA were measured at 0, 1, 2, and 3 hours; 7 days; and 3 months postoperatively. Pseudotesticular volumes and cosmetic appeal were assessed at 3 months.

**Result:**

Sixty-three patients were recruited (24 BSO, 21 BSCO, 18 BESO), 73% of whom were low-income earners. There was no significant difference in the procedure cost nor the PSA or testosterone nadirs achieved over the three-month follow-up period (11.3, 12.6, 15.2 ng/ml (*p*=0.667) and 0.44, 0.64, 0.79 nmol/l (*p*=0.603) respectively). Those with pseudotesticles (BSCO, BESO) felt less emasculated (*p*=0.010). BSCO produced the least sexual bother, highest sexual function, and largest pseudotesticular volumes. The cosmetic appeal scores were similar between groups (77.9 ± 22.8, 81 ± 13.9, and 81.9 ± 22.5, respectively, *p*=0.858).

**Conclusion:**

Endogenous pseudotesticular techniques, when compared with BSO, reduce the negative psychological impact experienced by patients without increasing costs. BSCO produced the best pseudotesticular volumes and postoperative sexual function. This study is registered with the ClinicalTrials.gov of the National Institute of Health U.S. National Library of Medicine as TEPSO study, NCT03744494: Comparison of the Therapeutic Efficacy and Patient Satisfaction of Three Techniques of Bilateral Orchidectomy in Prostate Cancer Patients of a Nigerian Sub-population. Registration completed on 16^th^ of November, 2018 (registered retrospectively) NCT03744494.

## 1. Introduction 

Prostate cancer (PCa) is the most prevalent cancer affecting Nigerian males [1]. Nigerian men with PCa commonly present late and thus require androgen deprivation therapy (ADT) [2, 3]. Regional reports from Nigeria indicate that 40%–51.3% of patients with PCa present with metastasis, and up to 94.2% have locally advanced disease at presentation [4, 5]. Despite the proven cost and survival benefits of surgical over medical castration [6–8], studies in other regions have shown either null difference or preference for medical castration over surgical castration [9–11]. Such preferences have been in a bid to avoid surgery or a negative body image due to an empty scrotum [8, 9, 12]. Although the patronage of medical options of castration in Nigeria is on the rise, bilateral orchidectomy is still the most common option of androgen deprivation used by Nigerian patients with advanced PCa due to the cheaper cost [2, 4, 5, 13]. It is therefore important to improve the quality of surgical castration available to Nigerian patients.

Varied documented forms of orchidectomy abound globally [14–16]. Endogenous or exogenous material could be used to create a pseudotesticle following orchidectomies. The use of exogenous testicular prostheses following bilateral orchidectomy for prostate cancer is not common worldwide [17]. Endogenous tissue could be derived from regional tissue such as the tunica albuginea, epididymis, or tunica vaginalis and used to refashion a pseudotesticle following removal of the testicle or its parenchyma. Two types of orchidectomy, bilateral simple and subcapsular orchidectomies without exogenous prostheses, have gained prominence in the Nigerian health sector [13, 18]. A study at Lagos, Southwestern Nigeria, suggested that the Nigerian patient would prefer bilateral subcapsular orchidectomy (BSCO) to avoid the psychological sequelae of an “empty scrotum” following bilateral simple orchidectomy (BSO), but this study did not actually compare its findings to those of patients who had BSO [19]. A later study in the same tertiary institution reported no significant difference in the quality of life of patients who had either BSO or BSCO and similar negative impact on body image [20]. Only one study has compared these with a third option of orchidectomy, bilateral-epididymal-sparing orchidectomy (BESO), amongst Indian patients with PCa [21]. In that study, BESO was the technique with the best cosmetic appeal. It was uncertain if the outcome would be similar among Nigerian men as race affects the size of the testis and its appendages [22].

This study aimed at comparing BSO, the practice at our centre, with two pseudotesticular techniques of orchidectomy (BSCO, practiced in some other centres in the country, and BESO) and evaluating the cosmetic appeal as perceived by the patient as well as the health-related quality of life (HRQoL). BESO has not been documented to have been used in African patients or compared with the more common forms of orchidectomy. Given racial differences in presentation, course, and response to therapy [23–25], it would be worthwhile to explore if BESO would be a viable option to improve the HRQoL of Nigerian men with PCa.

## 2. Methods

**Figure 1:**
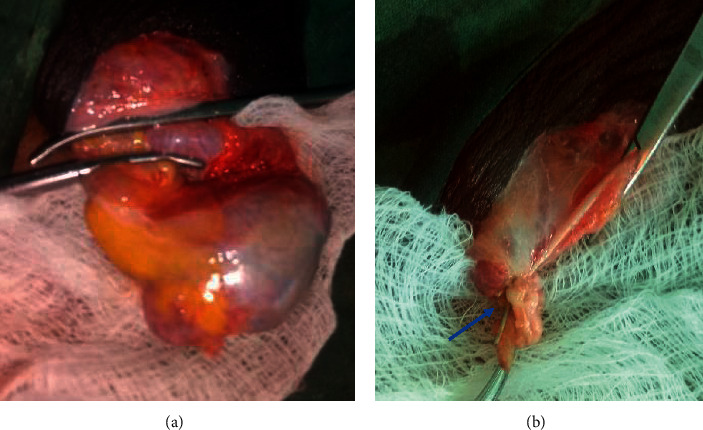
Simple orchidectomy. (a) Testicular vessels clamped. (b) Proximal testicular vascular stump ligated (arrow) and vas deferens clamped.

**Figure 2:**
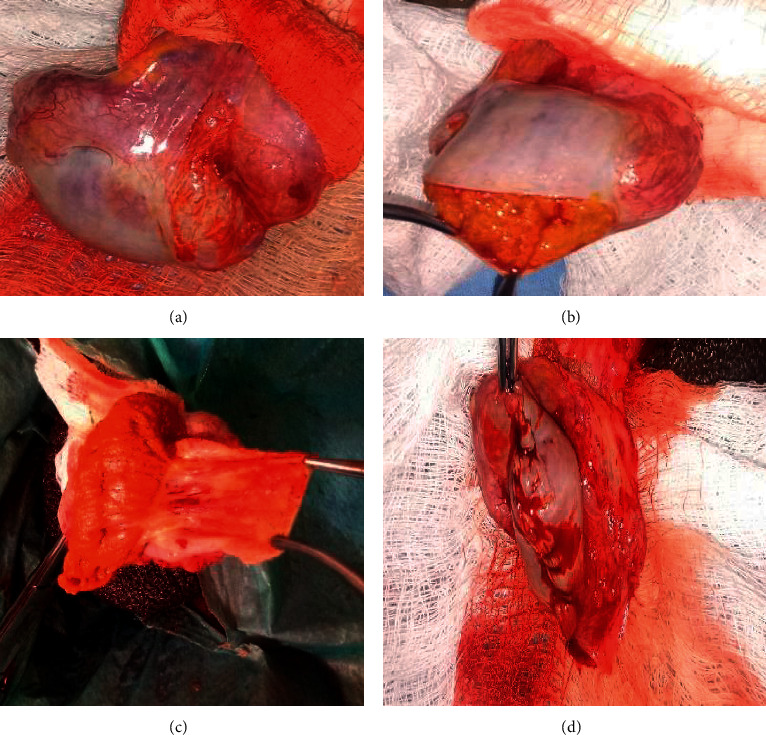
Subcapsular orchidectomy. (a) Vascular clamp applied. (b) Incised tunica albuginea. (c) Testicular parenchyma scraped off tunica albuginea. (d) Reapposed tunica albuginea to form a capsular pseudotesticle.

**Figure 3:**
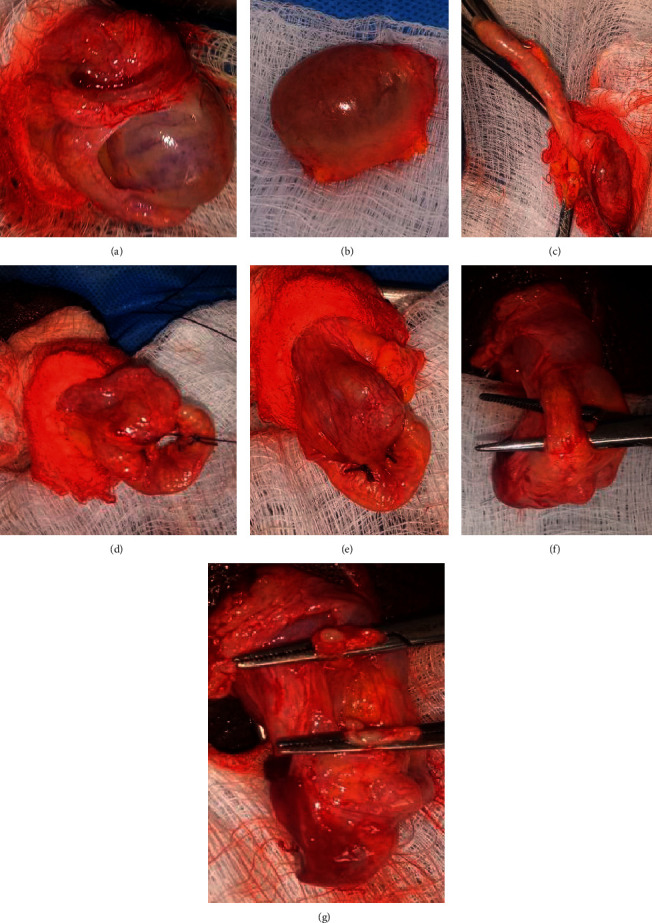
Epididymal-sparing orchidectomy. (a) Developed epididymal sinus. (b) Excised testis. (c) Isolated epididymis to be used as pseudotesticle. (d) Cauda of epididymis looped to meet the caput.(e) Epididymal pseudotesticle. (f) Isolated vas deferens. (g) Division of vas.

The cost benefit of orchidectomy was confirmed over the forms of medical castration in use at the centre prior to patient enrolment (Table 1). Proficiency in the pseudotesticular techniques was gained prior to patient recruitment. The study received ethical approval (UI/EC/15/0424) and was registered with ClinicalTrials.gov (Comparison of the Therapeutic Efficacy and Patient Satisfaction of Three Techniques of Bilateral Orchidectomy in Prostate Cancer Patients of a Nigerian Sub-population-TEPSO study, NCT03744494). Consenting patients who presented with locally advanced or metastatic PCa to the Division of Urology at the University College Hospital Ibadan and who opted for bilateral orchidectomy as ADT were recruited between March 2016 and November 2017. The patients were grouped into study arms, namely, BSO, BSCO, and BESO, via balloting (Figures 1–3). Clinical and sociodemographic information was obtained with a structured questionnaire and Expanded Prostate Cancer Index Composite (EPIC) demographic questionnaire (EPIC 2.2002). Preoperative assessments included HRQoL using the EPIC-26, Prader orchidometric volumes, serum prostate-specific antigen, and testosterone.

The surgeries (Figures 1–3) were done under local anaesthesia (0.5% xylocaine with adrenaline), with or without sedation (I.V. pentazocine with diazepam), via a median raphe incision. The spermatic cord structures were transected and ligated proximal to the testis and epididymis in BSO. In BSCO, a longitudinal incision was made on the tunica albuginea (TA), exposing the testicular parenchyma, which was cleared of it by pledget dissection. The hilum was ligated. Continuous interlocking sutures were used to appose the TA. In BESO, the epididymal sinus was developed, epididymal vessels ligated, and the testis excised. Epididymoplasty was achieved by folding the epididymis to juxtapose the epididymal head and tail, and consequent adjacent aspects of the body, which were sutured in place with continuous 3/0 vicryl sutures. The vas deferens was divided and ligated with 3/0 vicryl. The procedure was repeated on the contralateral testis. Layered closure was effected, and firm scrotal dressing and support were applied.

Repeat serum estimations of PSA and testosterone were taken on removal of the testicle or testicular parenchyma (0 hours), and at 1, 2, and 3 hours; 7 days; and 3 months postoperatively. Cosmetic appeal, HRQoL, and pseudotesticular volumes were assessed three months after surgery. A graduated visual analogue scale (VAS) rated from 0 to 100% was used for the patient's rating of the postoperative scrotal cosmetic outcome. EPIC-26 is a validated 26-item questionnaire with a 5-point Likert scale adapted from the UCLA-PCI (Prostate Cancer Index) to assess five domains: urinary incontinence, storage or voiding LUTS, bowel, vitality/hormonal, and sexual domains [26–28]. The sexual domain is subdivided into sexual function and bother [29]. The vitality/hormonal domains correlate with other questionnaires related to the assessment of depression and are used to make inferences [29]. EPIC-26 is unaffected by racial disparities [26]. Questions on bother were recoded, and all responses were rated from 0 to 100, with higher scores representing better HRQoL [30].

Data analysis was done with the Statistical Package for Social Sciences, version 20 (SPSS^®^ 20, IBM, UK) and STATA version 12 (StataCorp, LLC, US). Measures of central tendency and dispersion were used to analyse the quantitative parameters. The qualitative parameters were ranked. Variation in serum testosterone and PSA, sociodemographic and technical parameters, pre- and postoperative testicular volumes, cosmetic appeal, and HRQoL parameters were compared between the three groups and analysed using univariate analyses of variance (ANOVA) and multivariate analyses of variance, Student's *t*-test for the continuous, and Wilcoxon rank-sum test for the nonparametric measures. A repeated measures ANOVA with Greenhouse–Geisser correction was modelled for the average log testosterone and PSA concentration declines. A post hoc test and the least significant difference (LSD) were applied to the curves. The level of statistical significance, *p*, used was ≤0.05. The groups were dichotomised into those with and without pseudotesticles, and the associations were reevaluated.

## 3. Results

**Figure 4:**
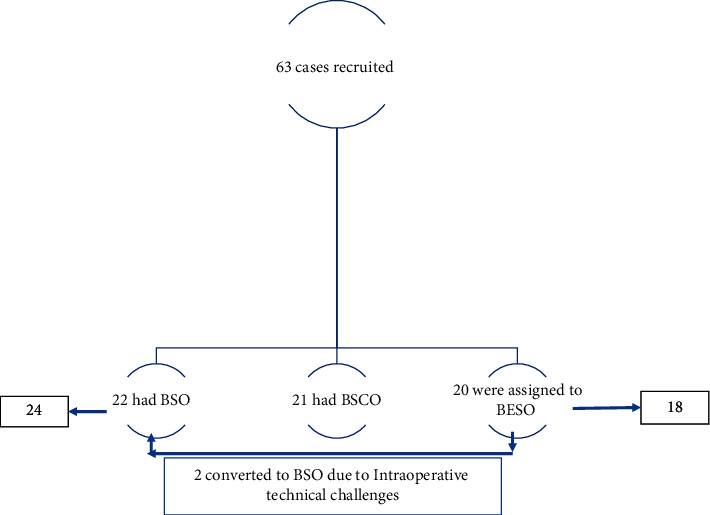
Schema of patient recruitment.

63 patients were recruited (Figure 4) and are summarised in Table 2. The mean operating time for all surgeries was 45 minutes. There was no significant difference in operating time or the need for supplementation with sedation between the orchidectomy categories (Table 3). None required cautery intraoperatively. The most common side-effect experienced was the hot flush. There was no significant difference in the incidence of complications (Table 3).

**Figure 5:**
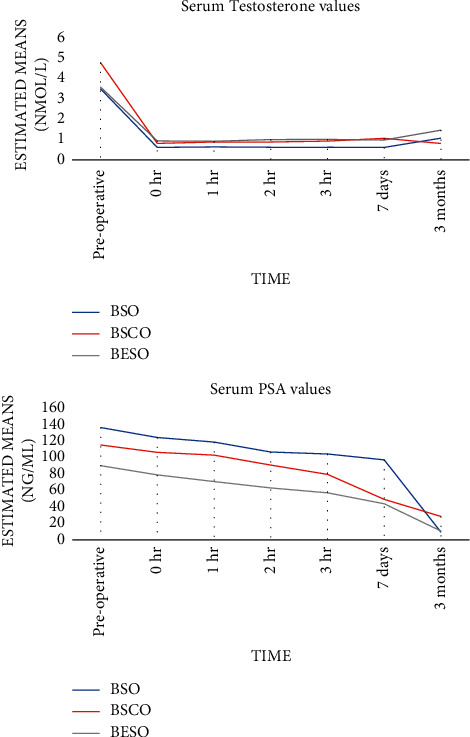
Serum testosterone and PSA trend over time (repeated measures ANOVA with Greenhouse–Geisser correction).

The baseline serum testosterone and PSA values for the BSO, BSCO, and BESO groups were 9.09 ± 11.11, 7.15 ± 11.09, 7.52 ± 7.18 nmol/l (*p*=0.160) and 173.92 ± 310.81, 79.82 ± 65.78 and 75.02 ± 45.45 ng/ml (*p*=0.122), respectively. At three months postoperatively, the mean testosterone was 1.09 ± 0.92, 0.73 ± 0.64, and 1.31 ± 0.62 nmol/l (*p*=0.294) and mean PSA was 19.45 ± 37.45, 23.47 ± 31.45, and 10.08 ± 6.35 ng/ml (*p*=0.623), respectively. There was no significant difference in the testosterone nadirs, time taken to achieve the nadir, and time to castrate levels (*p*=0.360, 0.979, and 0.085, respectively), or in the log testosterone curves between each surgical group (F: 1.12, *p*: 0.348). From 0 hours, the testosterone levels remained fairly static (Figure 5) but the median time to testosterone nadirs and surgical castrate levels was three hours. More of those in BSO category attained surgical castrate levels of ≤20 ng/ml (0.69 nmol/l), but this was not statistically significant, *p*=0.082. The average PSA levels differed between time points (F: 29.9, *p* < 0.001) but declines showed no significant difference in the interval PSA titres between each surgical group (F: 0.99, *p*: 0.395) as portrayed in Figure 5. The median time to PSA nadir was seven days, ranging between 0 hours and 3 months.

The summative QoL of all the patients in each category was similar before and after surgery but differed across domains. Those in the BSCO arm had the least sexual bother. There was no statistically significant effect of the type of orchidectomy on the changes observed in these domains. The surgical arms were regrouped into those without (Group I) and with (Group II) pseudotesticles. Sexual function was better among those with pseudotesticles (Table 4).

**Figure 6:**
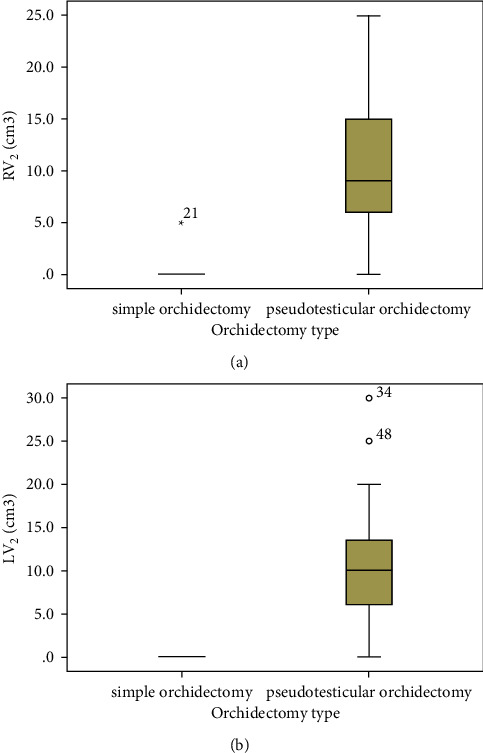
Comparison of the postoperative (a) RV_2_ and (b) LV_2_ pseudotesticular volumes.

**Figure 7:**
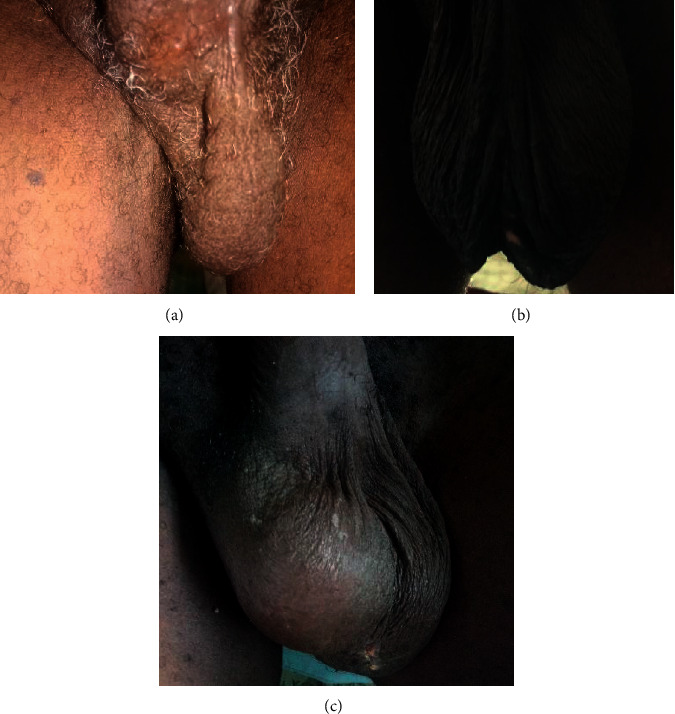
Initial postoperative photographs of patients who had (a) BSO, (b) BSCO, and (c) BESO.

**Figure 8:**
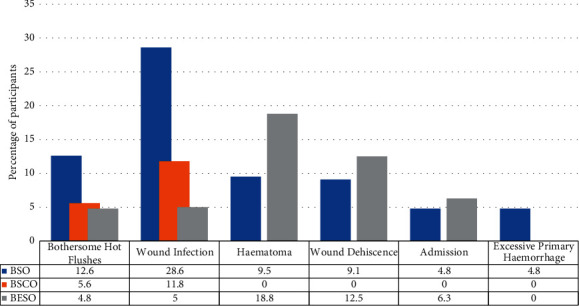
Complications of the patients in the different orchidectomy groups.

All surgery types caused varying degrees of decline in the postoperative pseudotesticular volumes (Figure 6), but BSCO produced the largest pseudotesticular volumes postoperatively (Table 5). Clinical photographs of the scrotal appearance of patients in each category are shown in Figure 7. Patients in BESO had larger immediate postoperative volumes by the visit on day 7 postoperatively (Figure 7(c)), but the volumes were not sustained and had declined three months postoperatively. The postoperative complications are shown in Figure 8.

More men felt a sense of emasculation after BSO (Table 6). Those with pseudotesticles felt less emasculated (*p*=0.010). On multivariate analysis, a sense of a compromised well-being was the only factor that remained independently related to sexual performance, which was better with pseudotesticles, and best with the BSCO category (*p* < 0.0001). On dichotomisation into Groups I and II, the sense of emasculation persisted as a drawback of BSO (Table 6). BSO participants were more likely to feel emasculated (OR: 2.54; CI_95_ : 1.18; 5.38; *p*=0.013). The sexual function was not significantly related to the measured pseudotesticular volume but rather lower in those who perceived their testicles as smaller or felt emasculated. Using a four-item regression model (well-being compromise, sense of emasculation, sexual function, sexual bother), lower cosmetic appeal satisfaction scores were associated with a sense of compromised well-being (adjusted *R*^2^ = 0.071; CI_95:_ 2.3; 58.0; *p*=0.035).

## 4. Discussion

The three types of orchidectomy studied were therapeutically equivalent in terms of PSA and testosterone reduction. Agarwal and Agarwal [21] demonstrated equivalent PSA and testosterone declines in patients who had BSO, BSCO, or BESO while some other studies have shown therapeutic equivalence between BSO and BSCO, [14, 20, 31] or BSO and BESO [32], thus buttressing the fact that the pursuit of cosmetic appeal did not take precedence over effective PCa therapy.

Pseudotesticular techniques did not add significantly to the cost of care in terms of theatre time, suture, sedation, or cautery requirements. Interestingly, BSO required more sutures and different suture strengths to ligate the vessels and complete the surgery, so it was potentially more expensive. A single suture strength was used for BSCO and BESO. A diabetic patient who had BSO served as an outlier, with four complications, but this did not result in a significantly higher average number of complications. BSCO has been found to have a higher complication rate and procedure time than BSO [20, 32], but this was not the case in our study. It could be argued that the lack of prior familiarity with the pseudotesticular techniques led to greater care with them or that the months taken to improve proficiency in the two techniques (BSCO and BESO) techniques before study commencement were beneficial. Overall cost considerations were important outcomes as most of the patients were retirees, and 73% earned less than ₦ 2,740 ($7.60 USD) per day when derived from the annual earning category of less than ₦ 1,000,000 per year used in the questionnaire. It could not be ascertained whether their earnings actually fell below the poverty line of $1.25 per day, as the exact earnings were not determined. It was our desire to identify a more palatable yet cheaper choice of orchidectomy.

Preoperatively, the patients' QoL, including sexual function, was similar between the three groups. The worsening sexual function postoperatively was significant, with BSO having the least and BSCO the highest sexual function scores. Those with pseudotesticles also had less sexual bother. The sexual function scores were not related to their relationship status, lower testosterone nadirs of BSO, or the baseline testosterone levels. Our patients had locally advanced or metastatic disease. None had “very good” sexual function preoperatively. 24 (38%) preoperatively and 7 (11%) postoperatively had either “fair” or “good” sexual performance. This may explain why testosterone levels were not significantly related to the sexual function declines. The importance of potency preservation has been emphasised in the treatment of PCa as men have been shown to choose sexual potency over a longer life-span [33], and the finding of better sexual function in this study in patients with pseudotesticles may buttress a need to employ these techniques. Orakwe et al. [20], in another centre in Nigeria, did not find any significant difference in QoL, but this may be due to a difference in the domain construct of the QoL instrument used (FACT-P and FACT-G) and the domains assessed. Similarly, Singh et al. [34] in India did not report significant differences in the QoL of patients randomised to BSO and BSCO, assessed using the Modified Fugl-Meyer questionnaire, interrogating social, sexual, vocational, and financial domains. In that study, the majority did not report psychometric deficits attributable to scrotal contents either. The Prostate Cancer Outcomes Study premised a loss of libido and erectile dysfunction on ADT-induced reduction in testosterone levels [27]. Not only was this a much larger study with 431 patients, but also 52.1% of the men had T1 or T2 disease, which differs in clinical presentation from locally advanced disease. Locally advanced disease is more likely to present with erectile dysfunction from infiltration of the nervi erigentes, poor libido, and poor sexual performance before ADT.

The patient's perception of testicular volume, rather than the actual pseudotesticular metrics, was associated with higher satisfaction scores. Better satisfaction scores reduced the feeling that their well-being had been compromised by the surgery. Those with pseudotesticles felt less emasculated and less compromised (BSCO > BESO > BSO). In comparing the perceived sense of emasculation, BSO resulted in a higher sense of emasculation than BSCO among another Nigerian cohort of men with PCa [20]. The only study found in English literature, which compared the three orchidectomy categories, showed that BESO had the highest satisfaction scores, followed by BSCO [21]. This may be because BESO has the potential to produce pseudotesticles described as even larger than the native testicles [35]. Orchidometric volumes were not assessed in the study by Agarwal and Agarwal [21] to provide objective evidence of larger pseudotesticular volume with BESO. In addition, Asians have been shown to have smaller testes, seminiferous tubular volume, and breadth, which may result in smaller epididymides; thus, the epididymoplasty may have been sufficient to create a pseudotesticle similar in volume to the relatively smaller native testis. This may not be the case for men of African descent. Mean left and right testicular volumes in TEPSO were 17.5 ± 6.9 and 19.2 ± 7.6 while those in an Asian population were 14.2 ± 1.3 and 13.3 ± 1.2, respectively [36]. Although our patients in the BESO category had larger pseudotesticles initially, this was not sustained. This may be due to androgen deprivation-related apoptosis of, most significantly, the principal cells of the epididymis documented to start between two and four months postorchidectomy, and to stabilise by a year afterwards [35, 37]. The additional intracapsular haematoma consolidation and consequent fibrosis in BSCO may explain the larger pseudotesticles in this category.

## 5. Limitations

Prader orchidometer beads have fixed volumes and curvilinear shapes. Pseudotesticles are not perfectly curvilinear. Thus, the bead volumes documented were estimations of pseudotestiular volumes. A sonographic volumetric measurement may have been more accurate but would have added to the cost of the study [16]. Testosterone has a circadian rhythm with a peak at 8 a.m. daily, so preoperative values and values on day 7 and three months postoperatively were taken by this time [38]. Patient attrition or mortality by the three-month assessment reduced the power of the results obtained. The EPIC-26 assessment of the urinary domain has no provision for those with a urethral catheter, leading to nonresponses in LUTS-related questions.

## 6. Conclusion and Recommendations

The three surgical techniques are equivalent in therapeutic efficacy. The presence of a pseudotesticle reduces the sense of emasculation felt by men following bilateral orchidectomy. This reduction in psychometric deficits comes with no increase in cost incurred or complications encountered. BSCO produced better pseudotesticular volumes than BESO by the three-month-postoperative assessment. Those in the BSCO category had the least sexual bother and the best sexual function. Endogenous pseudotesticular techniques, preferably BSCO, should be used following bilateral orchidectomy to reduce the negative psychological impact on the patients.

## Figures and Tables

**Table 1:** Costs of various hormonal therapies for prostate cancer, in and around UCH Ibadan (informal market survey in 2015^∗∗^ and 2018 ^).

Treatment	Cost per patient (₦)	SOP cost (₦)	Recurrent costs per year	Total cost per year (₦)	Total cost by 30-month minimum treatment period (₦)	Cost of serum testosterone assay (₦)	Cost of serum PSA assay (₦)	Total cost per year (₦)
Orchidectomy in the main theatre under LA^∗∗^	39,500	12,500	No	39, 500 or 12, 500^*∗*^	39, 500 or 12, 500	4000 (+4000 post-op)	3,000 (x2 = 6000 per year)	66,000 or 26, 500 first year; 6000 in subsequent years
Orchidectomy in the main theatre under LA^	83,500	22,500	No	83, 500 or 22, 500^*∗*^	83, 500 or 22, 500	4500 (+4500 post-op)	3,500 (x2 = 7,000 per year)	99,500 or 38,500 first year; 7,000 in subsequent years
SC goserelin (monthly dose)^∗∗^	18,000	—	Yes	216, 000	540, 000	4000 (x3-4 = 12,000–16,000 per year)	3,000 (x2 = 6,000 per year)	238,000
SC goserelin (monthly dose)^	56,000	—	Yes	672, 000	1,680,000	4500 (x3-4 = 13,500–18,000 per year)	3,500 (x2 = 7,000 per year)	692,500–697,500
SC goserelin (3 monthly doses)^∗∗^	54,000	—	Yes	216,000	540,000	4000 (x3-4 = 12–16,000 per year)	3,000 (x2 = 6000 per year)	238,000
SC goserelin (3 monthly doses)^	85,000–110,000	—	Yes	340,000–440,000	2,550,000–3,300,000	4500 (x3-4 = 13,500–18,000 per year)	3,500 (x2 = 7,000 per year)	360,500–465,000
Bicalutamide (monthly dose)^∗∗^	24,000	—	Yes	288,000	720,000	4000 (x3-4 = 12,000–16,000 per year)	3,000 (x2 = 6000 per year)	319,000
Bicalutamide (monthly dose)^	28,000	—	Yes	336,000	840,000	4500 (x3-4 = 13,500–18,000 per year)	3,500 (x2 = 7,000 per year)	356,500–361,000

₦360: 1 USD. ^*∗*^Some of the patients may benefit from the use of the surgical outpatient (SOP) theatre, at cheaper rates. LA : local anaesthetic; SC : subcutaneous.

**Table 2:** Sociodemographic characteristics of subject participants.

	BSO	BSCO	BESO	*P* value
Age, mean (SD)	69.7 (8.4)	70.9 (12.0)	70.2 (8.7)	0.880

Relationship status, N (%)				0.761
Living with spouse	17 (70.8)	13 (72.2)	13 (72.2)	
Significant relationship	3 (12.5)	3 (14.3)	2 (11.1)	
Single	2 (8.3)	2 (9.5)	2 (11.1)	
Widower	2 (8.3)	3 (14.3)	1 (5.6)	

Work status, N (%)				0.984
Unemployed	1 (4.2)	1 (4.8)	0 (0.0)	
Part time	3 (12.5)	1 (4.8)	1 (5.6)	
Full time	2 (8.3)	3 (14.3)	2 (11.1)	
Retired	18 (75.0)	16 (76.2)	15 (83.3)	

Educational status, N (%)				0.219
Primary or less	8 (33.3)	9 (32.8)	5 (27.8)	
Some high/technical	4 (16.7)	3 (14.3)	3 (16.7)	
Secondary	4 (16.7)	2 (9.5)	6 (33.3)	
Some university	3 (12.5)	3 (14.3)	1 (5.6)	
University graduate	1 (4.2)	4 (19.0)	3 (16.7)	
Postgraduate	4 (16.7)	4 (19.0)	0 (0.0)	

Annual combined household income (₦), N (%)				0.356
<1,000,000	17 (70.8)	15 (71.4)	14 (77.8)	
1,000,000–3,000,000	6 (25.0)	3 (14.3)	4 (22.2)	
3,000,001–10,000,000	1 (4.2)	0 (0.0)	17 (94.4)	
More than 10,000,000	0 (0.0)	3 (14.3)	0 (0.0)	

Average Gleason score	7.8	7.6	8.4	0.133

**Table 3:** Summary of perioperative events.

Technical parameters	BSO	BSCO	BESO	*P* value
Mean operating time (SD) in minutes	46.65 (7.8)	44.62 (16.7)	46.9 (11.1)	0.293
Sedation required, frequency (%)	13 (54.2)	11 (52.4)	9 (50.0)	0.938
Mean suture number, mean (SD)	2.9 (0.8)	2.6 (0.7)	2.6 (0.5	0.215
Number of complications, mean (SD)	0.86 (0.9)	0.35 (0.8)	0.43 (0.8)	0.118

^*∗*^Independent samples *t*-test used.

**Table 4:** The mean (SD) postoperative EPIC-26 scores of those without (Group I) and with (Group II) pseudotesticles.

	Preoperatively			Postoperatively		
	Group I	Group II	*P* value	Group I	Group II	*P* value
Urinary incontinence	64.44 (29.61)	73.76 (25.99)	0.283	68.36 (32.14)	85.56 (20.81)	0.096
Urinary LUTS	81.25 (17.42)	77.42 (23.20)	0.547	80.80 (21.01)	85.00 (21.59)	0.577
Bowel domain	83.73 (11.36)	80.4 (17.63)	0.464	94.79 (7.53)	90.34 (13.75)	0.250
Sexual function	22.05 (26.70)	27.13 (29.36)	0.529	9.41 (18.65)	30.54 (30.44)	**0.019**
Sexual bother	59.52 (36.64)	62.50 (42.12)	0.792	59.72 (38.48)	67.86 (35.48)	0.497
Hormonal/vitality domain	82.14 (19.91)	82.5 (17.51)	0.946	90.00 (15.81)	90.48 (10.94)	0.913

**Table 5:** Comparison between the testicular and pseudotesticular volumes from the different orchidectomies (significant differences in volume are emboldened).

Orchidometric volume	BSO	BSCO	BESO
RV1	20.2 (8.9)	18.1 (6.7)	18.2 (6.7)
RV2	0.2 (1.0)	10.3 (4.7)	8.3 (8.1)
*p* value (RV2–RV1)	**0.001**	**0.011**	**0.001**

LV1	19.6 (8.5)	15.6 (9.7)	15.7 (5.8)
LV2	0.0 (0.0)	6.5 (8.2)	5.8 (6.9)
*p* value (RV2–RV1)	**0.001**	0.216	**0.022**

RV1: preoperative right testicular volume; RV2: postoperative right pseudotesticular volume; LV1: preoperative left testicular volume; LV2: postoperative pseudotesticular volume.

**Table 6:** Comparison of patient satisfaction with surgery done.

	BSO	BSCO	BESO	*P* value
Satisfaction score, mean (SD)	77.9 (22.8)	81.0 (13.9)	81.9 (22.5)	0.858
I feel my testes are smaller postop, N (%)	13 (72.2)	8 (88.9)	7 (53.8)	0.360
I feel less like a man, N (%)	11 (64.7)	3 (33.3)	3 (23.1)	0.036
I feel my well-being has been compromised, N (%)	3 (16.7)	0 (0.0)	2 (15.4)	0.363

	Group I	Group II		*P* value
Satisfaction score, mean (SD)	77.9 (22.8)	81.52 (18.9)		0.584
I feel my testes are smaller postop, N (%)	12 (70.6)	15 (68.2)		0.654
I feel less like a man, N (%)	11 (68.8)	6 (27.3)		0.010
I feel my well-being has been compromised, N (%)	3 (16.7)	2 (8.3)		0.182

Group I: no pseudotesticles; Group II: pseudotesticles (BSCO and BESO).

## Data Availability

The data and methods are available from the corresponding author, on request, with the stated reason(s). The study protocol is available at ClinicalTrials.gov, the NIH registry https://clinicaltrials.gov/ct2/show/study/NCT03744494.
